# The Development and Use of a New Visual Tool (REVISIT) to Support Participant Recall: Web-Based Interview Study Among Older Adults

**DOI:** 10.2196/52096

**Published:** 2024-02-01

**Authors:** Eileen M Dryden, Chitra Anwar, Jennifer Conti, Jacqueline H Boudreau, Meaghan A Kennedy, William W Hung, Kathryn A Nearing, Camilla B Pimentel, Lauren Moo

**Affiliations:** 1 Center for Healthcare Organization and Implementation Research VA Bedford Healthcare System Veterans Health Administration Bedford, MA United States; 2 New England Geriatric Research, Education, and Clinical Center VA Bedford Healthcare System Veterans Health Administration Bedford, MA United States; 3 Chobanian & Avedisian School of Medicine Boston University Boston, MA United States; 4 Bronx Geriatric Research, Education, and Clinical Center James J. Peters VA Medical Center Veterans Health Administration Bronx, NY United States; 5 Icahn School of Medicine New York, NY United States; 6 Eastern Colorado Geriatric Research, Education, and Clinical Center Rocky Mountain Regional VA Medical Center Veterans Health Administration Aurora, CO United States; 7 Division of Geriatric Medicine University of Colorado Anschutz Medical Campus Aurora, CO United States; 8 Department of Public Health Zuckerberg College of Health Sciences University of Massachusetts Lowell Lowell, MA United States; 9 Harvard Medical School Boston, MA United States

**Keywords:** qualitative interviews, visual recall aid, older adults, health services research, web-based methods, visual tool, recall, qualitative interview, experience, perspective, motivation, patient, recall capacity, medical information, visual appointment, geriatric, older people, telemedicine, videoconference, e-consultation, e-medicine, internet medicine, REVISIT, Remembering Healthcare Encounters Visually and Interactively, mobile phone

## Abstract

**Background:**

Qualitative health services research often relies on semistructured or in-depth interviews to develop a deeper understanding of patient experiences, motivations, and perspectives. The quality of data gathered is contingent upon a patient’s recall capacity; yet, studies have shown that recall of medical information is low. Threats to generating rich and detailed interview data may be more prevalent when interviewing older adults.

**Objective:**

We developed and studied the feasibility of using a tool, Remembering Healthcare Encounters Visually and Interactively (REVISIT), which has been created to aid the recall of a specific telemedicine encounter to provide health services research teams with a visual tool, to improve qualitative interviews with older adults.

**Methods:**

The REVISIT visual appointment summary was developed to facilitate web-based interviews with our participants as part of an evaluation of a geriatric telemedicine program. Our primary aims were to aid participant recall, maintain focus on the index visit, and establish a shared understanding of the visit between participants and interviewers. The authors’ experiences and observations developing REVISIT and using it during videoconference interviews (N=16) were systematically documented and synthesized. We discuss these experiences with REVISIT and suggest considerations for broader implementation and future research to expand upon this preliminary work.

**Results:**

REVISIT enhanced the interview process by providing a focus and catalyst for discussion and supporting rapport-building with participants. REVISIT appeared to support older patients’ and caregivers’ recollection of a clinical visit, helping them to share additional details about their experience. REVISIT was difficult to read for some participants, however, and could not be used for phone interviews.

**Conclusions:**

REVISIT is a promising tool to enhance the quality of data collected during interviews with older, rural adults and caregivers about a health care encounter. This novel tool may aid recall of health care experiences for those groups for whom it may be more challenging to collect accurate, rich qualitative data (eg, those with cognitive impairment or complex medical care), allowing health services research to include more diverse patient experiences.

## Introduction

Qualitative health services research often relies on semistructured or in-depth interviews to develop a deeper understanding of patient experiences, motivations, and perspectives. The quality of data gathered is contingent upon a patient’s recall capacity. Studies consistently show recall of medical information is low. Patients remember between 20% and 60% of the information provided by health care practitioners immediately after an encounter [1], dropping to 12.8% a month later [2]. In a seminal study of patient recall in a routine clinical setting by Anderson et al [3], of the 40% of medical information recalled by patients, 48% of it was misconstrued. Various practitioner- and patient-related factors pose threats to recall: practitioner-related factors include the use of complicated medical terminology, high volume of information relayed, and mode of information presentation (eg, verbal vs visual), while patient-related factors include low education level and emotional state during the visit [1,4].

Threats to generating rich and detailed interview data may be more prevalent when interviewing older adults. Aging is associated with a decline in sensory and cognitive function, making it difficult to understand and remember medical information [5]. Compared to younger individuals, older adults have more difficulty recalling details of health care experiences that researchers may be interested in exploring, including medication regimens [6], treatment recommendations [7], and appointment reminder telephone messages [8]. Routine recurring visits are also more poorly recalled than nonrecurring ones—patients tend to collapse recurring visits into a single, generic memory instead of separate, specific occurrences [9]. Older adults may be especially prone to do so as they are estimated to have an average of 7 medical visits per year [10].

To ensure qualitative data are accurate, researchers must carefully consider how to plan and conduct qualitative interviews with older adults. Visual methodologies have been used to mitigate the threats to validity resulting from recall bias in qualitative health services research [11-13]. These methods invite participants to tap into memories through nonverbal ways of thinking, improving participant recall and allowing researchers to access participant perspectives that can be difficult to articulate through conversation alone. Commonly used strategies include viewing and discussing photographs, video elicitation, drawing, chart-stimulated recall, and mapping and timelining exercises [11,14,15]. In our review of the literature, we found no documented cases of using visual recall aids with older adults, a group for whom such tools may be particularly useful, given known challenges with medical information recall [1].

In this paper, we explore the development and use of a new visual tool, Remembering Healthcare Encounters Visually and Interactively (REVISIT), created to aid recall of a specific telemedicine encounter among older adult interview participants. In spring 2021, a team of Veterans Health Administration (VA) qualitative researchers interviewed 30 rural, older (65 years of age and older) veterans and their caregivers remotely as part of an evaluation of GRECC Connect, a program that uses telemedicine to connect rural veterans with complex care needs to geriatric specialty care at 15 urban VA medical center hub sites. GRECC Connect hub teams are comprised of interprofessional care teams affiliated with Geriatric Research, Education, and Clinical Centers, VA centers of excellence focused on aging. Given the focus of many GRECC Connect sites on treating cognitive impairment, we anticipated that interviewees might experience challenges recalling details of their most recent GRECC Connect appointment (the “index visit”), posing a risk to the completeness and validity of interview data. We also anticipated challenges isolating information about their most recent GRECC Connect appointment from other appointments due to the increase in telemedicine visits during the COVID-19 pandemic. The REVISIT visual appointment summary was developed to better facilitate interviews conducted remotely with our participants. Our primary aims were to aid participant recall, maintain focus on the index visit, and establish a shared understanding of the visit between participants and interviewers. In this paper, we describe the development of REVISIT and interviewer experiences with the tool and suggest considerations for broader implementation and future research to expand upon this preliminary work.

## Methods

### Evaluation Team

A multidisciplinary VA project team contributed to the evaluation. Team members included physicians with expertise in primary care, geriatrics, and dementia; a veteran consultant; GRECC Connect leadership; and researchers with expertise in qualitative methods and project coordination.

### Developing REVISIT

REVISIT was designed as a template to be populated with data from the veteran’s electronic health record (EHR). A member of the team with a background in media design drafted template options on Canva (Canva), a free web-based graphic design platform. REVISIT’s design drew upon VA’s Patient Experience Journey Map, a visual representation of commonly experienced moments before, during, and after a veteran’s health care visit [16]. Draft REVISIT templates were presented to the full multidisciplinary team for review, resulting in 3 iterative rounds of feedback and refinement.

The information included in the final iteration of REVISIT focused on aspects of the index visit we sought to confirm and explore, which were separated into three groupings: (1) the referral, including the reason and referring provider; (2) the index visit, including individuals present and main topics discussed; and (3) changes in the veteran’s health and health care resulting from the visit, including changes in diagnoses, medications, and referrals.

**Figure 1:**
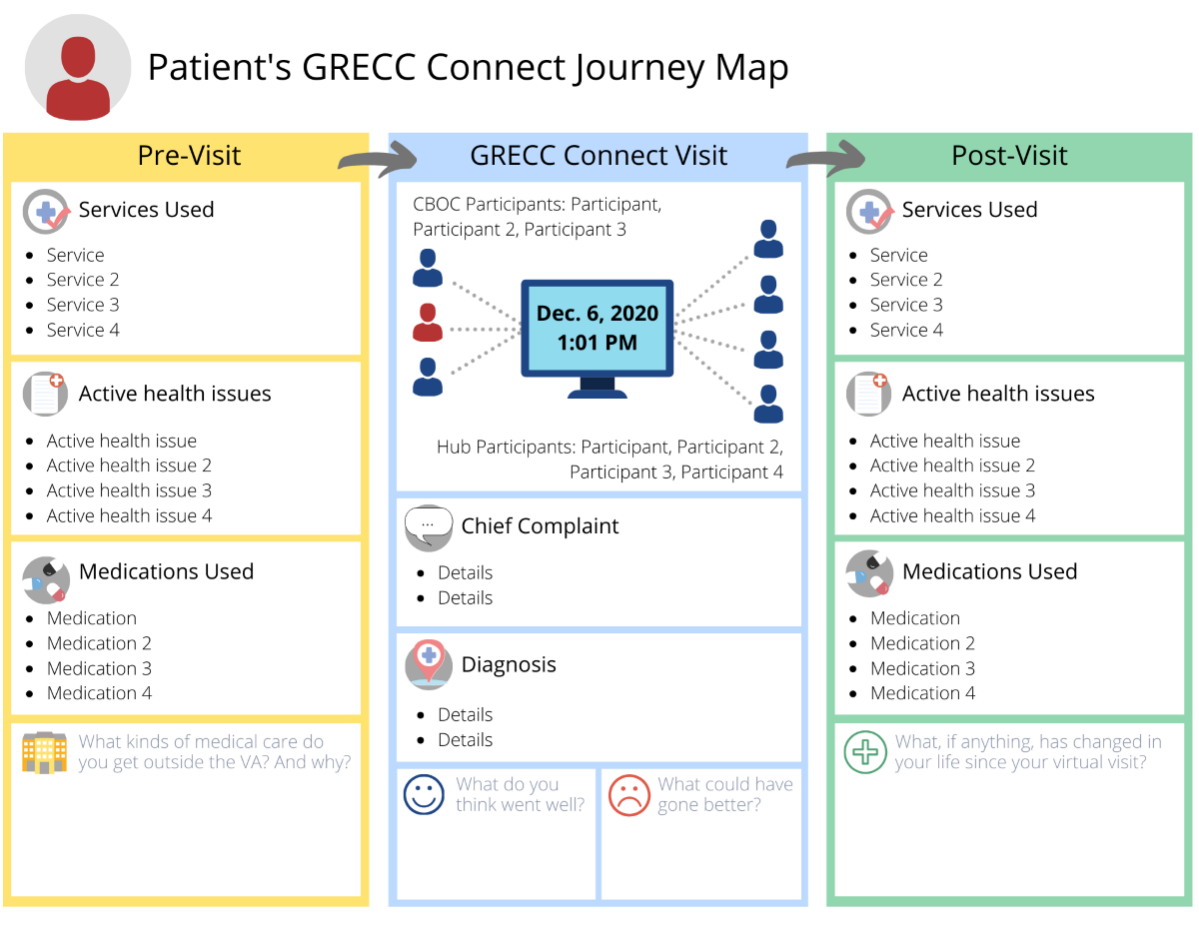
First iteration: the first iteration of REVISIT included elements subsequently omitted, such as the sections containing questions at the bottom of each column. REVISIT: Remembering Healthcare Encounters Visually and Interactively.

The overall structure of the first iteration of REVISIT (Figure 1) contained 3 columns, with each column representing a step in the GRECC Connect visit (before, during, and after the visit). For the first iteration, initial refinements suggested by the team focused on simplifying the template to include only information pertinent to the interview. Team members also felt REVISIT should focus more on the “Post-Visit” section to better aid participants’ recall of what worked well about the visit and what health needs remained unmet.

**Figure 2:**
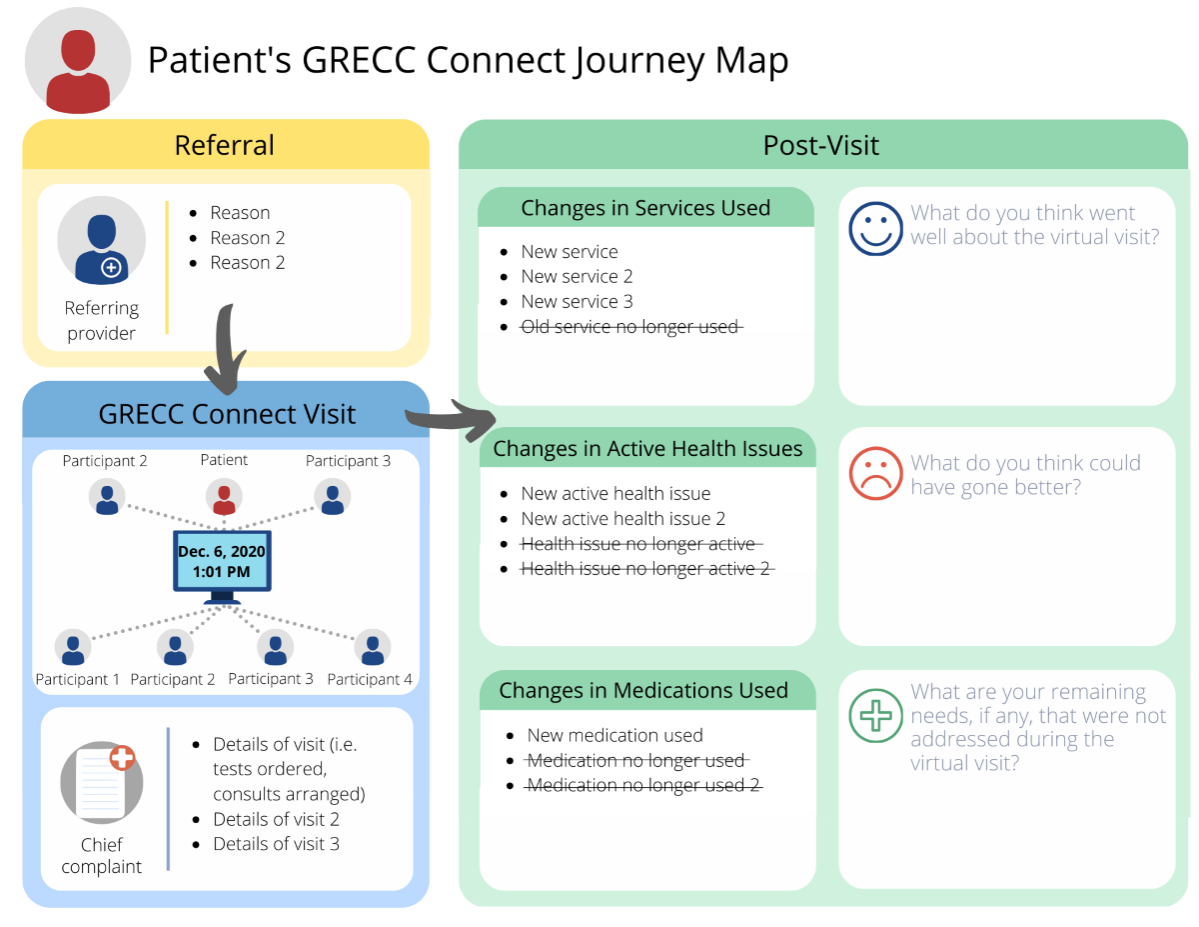
Second iteration: the second iteration of REVISIT includes an expanded “Post-Visit” section and simplification of the other 2 sections. REVISIT: Remembering Healthcare Encounters Visually and Interactively.

The second iteration (Figure 2) incorporated the aforementioned feedback for simplification. For example, the “Pre-Visit” section was changed completely to include only GRECC Connect referral information, and the heading for this section was edited to “Referral” to reflect this change. The “GRECC Connect Visit” section still included persons present during the index visit, but the other sections were collapsed into one summary of the visit details. The “Post-Visit” section was expanded to take up more of the page, emphasizing this section as the focus of the interview.

After reviewing the second iteration, the team encouraged further simplification of the template’s design to reflect REVISIT’s primary goal of helping participants recall details about their experience of the telemedicine visit. Team review of the second iteration also focused on possible modifications to the included language. EHRs contain medical jargon that is likely unfamiliar to interview participants. Team members suggested translation of these terms into more lay language for the last iteration, a process that relied heavily on input from the physician team members.

**Figure 3:**
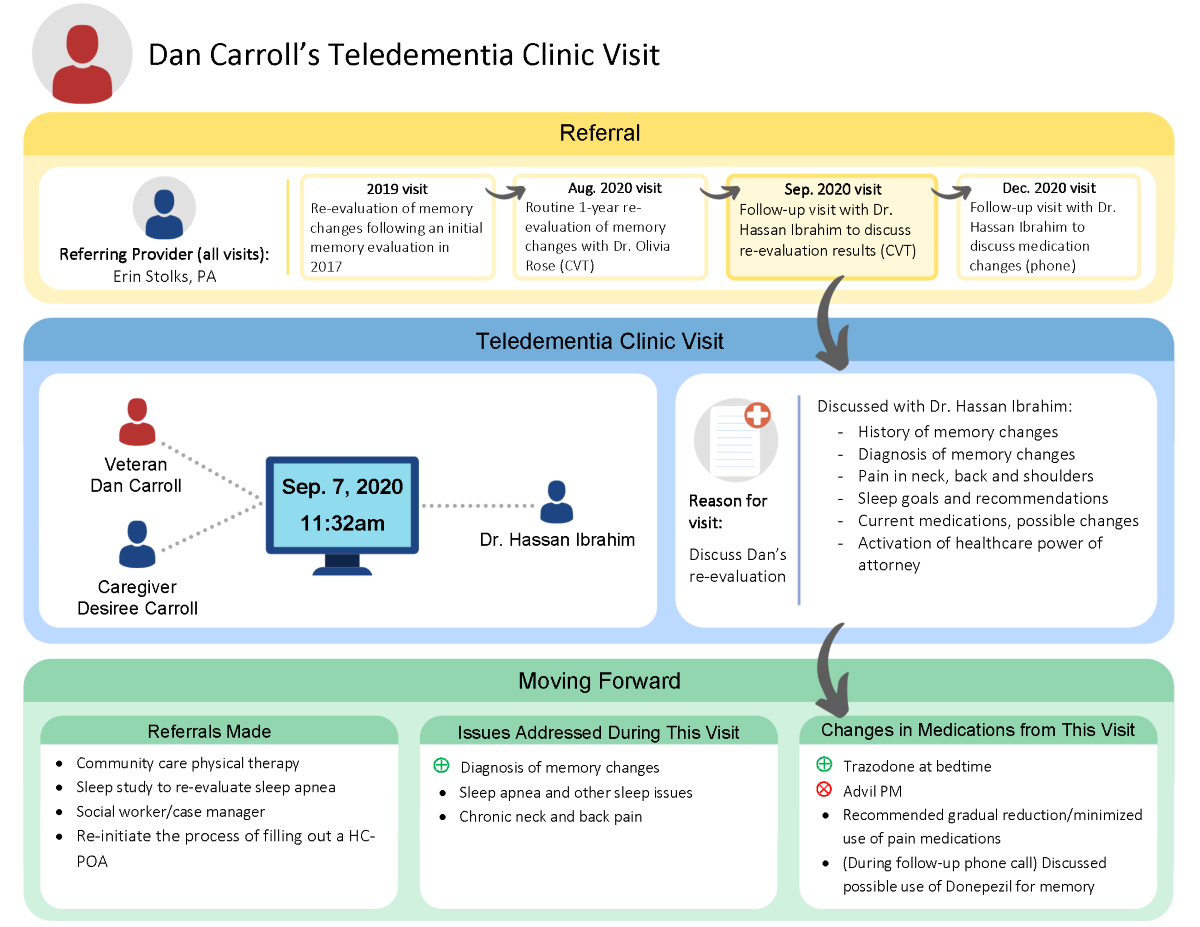
Final iteration: the final iteration of REVISIT includes edits to language used, such as “memory changes” instead of specific diagnoses, and “Moving Forward” instead of a “Post-Visit” heading. This version also reflects the omission of interview probe questions in the “Moving Forward" section. This sample REVISIT includes only fictional participant information. REVISIT: Remembering Healthcare Encounters Visually and Interactively.

Design considerations for the final REVISIT iteration (Figure 3) included using boxes with rounded edges, as the team felt this connoted friendliness compared to the sharp edges shown in Figure 1. Icons were included alongside text descriptions wherever possible to increase ease of understanding. Arrows showed flow from one section to the next, green “+” symbols signified newly prescribed medications, and red “x” symbols signified deprescribed medications. Calibri font was used in accordance with VA’s graphic design standards.

The colors of each section were specifically chosen based on color-in-context theory [17], which posits color meanings are grounded in learned associations that develop from repeated pairings of colors with particular messages, concepts, or experiences. The color motif was loosely based on the 3 phases of a traffic light—the initial referral to GRECC Connect was yellow (to symbolize a transition) and the postvisit was green (to symbolize moving forward). Blue, as opposed to red, was chosen to represent the index visit because of its generally accepted calming effects [18]. The team felt this was a more suitable color choice, given the potentially sensitive topics that may surface during discussion of the index visit during interviews. The team also opted for colors with lighter versus darker hues, as these were felt to be easier on the eyes.

Language edits were incorporated into the final iteration. Potentially sensitive medical issues such as dementia diagnoses or cognitive decline were instead referred to as “memory changes.” We deemed this step necessary as it was sometimes unclear, based on the medical notes, what was explicitly discussed with the participant versus only documented in the clinic note. The inclusion of more neutral language helped ensure that REVISIT would avoid distressing a participant with potentially new information about their health.

### Participants

REVISIT was used in the context of a health care evaluation with a sample of 30 rural veterans attending specialty geriatric telemedicine visits at 6 geographically diverse GRECC Connect hub sites. We defined “telemedicine” as medical appointments conducted through one of three modalities: (1) video appointments from a veteran’s home or other location to a remote specialist using VA Video Connect (VVC), (2) video appointments from a VA outpatient clinic near the veteran’s home to a remote specialist using Clinical Video Telehealth (CVT), or (3) telephone. An option to participate as a veteran-caregiver dyad was offered in cases where veteran participants had some degree of cognitive impairment or where caregivers were substantially involved in care. Due to the impact of dementia, some dyads were primarily represented by the caregiver.

Prior to initial contact, 3 team members briefly reviewed each veteran’s EHR to confirm the most recent telemedicine visit date and modality (VVC, CVT, or phone), veteran location, initial reason for referral, the presence of a caregiver, and any cognitive or other health concerns that would preclude study participation (eg, a veteran in hospice or deceased). Veterans were considered eligible for participation if they were 65 years and older of age, resided in a rural area (rural-urban commuting area [RUCA] >1), participated in a telemedicine GRECC Connect appointment between December 2020 and March 2021, and spoke English as their primary language. Verbal permission was obtained from each veteran and caregiver to participate in the evaluation.

### Interview Preparation

Once participants agreed to participate in an interview, a team member performed a detailed chart abstraction of the veteran’s EHR 6 months prior to the index appointment using a structured data abstraction template (Multimedia Appendix 1). This data abstraction template helped team members extract only information relevant to the GRECC Connect visit from the participant’s EHR, which can contain many notes from numerous clinicians. GRECC physicians on the multidisciplinary team helped to develop the data abstraction template and interpret EHR data when questions arose. These data were then used to populate REVISIT to create an individualized visual appointment summary and tailored interview guide for each participant. To protect participants’ health information, each populated REVISIT was saved in a password-protected participant-specific folder on a secure server. On average, team members spent 2 to 4 hours abstracting data and creating the visual summary. Time varied depending on the extensiveness and clarity of the participant’s medical chart.

### Data Collection

Four experienced qualitative researchers conducted semistructured qualitative interviews with veterans and their caregivers who agreed to participate in the evaluation. We asked participants about their GRECC Connect telemedicine visit, including support received, what worked and did not work well, preferred modality for medical care, impact of visit, satisfaction, and recommendations. Interviews took place approximately a month after the index visit and were conducted via VVC or by phone depending on participant preference and ability. In total, 16 interviews were conducted via VVC on various devices (eg, smartphone, tablet, and laptop), and 14 were conducted by phone.

REVISIT was shared with participants who were interviewed via VVC using its screen-sharing feature. The use of REVISIT was incorporated into the GRECC Connect interview guide. The interviewer shared REVISIT when beginning to discuss the index visit following initial rapport-building questions. In at least one case, REVISIT was shown earlier in the interview because the participant needed more recall support.

Following the data collection process, team members who conducted interviews debriefed their experience using REVISIT, sharing the benefits and challenges of using the tool. Elements of the debrief were recorded on digital sticky notes, which were then grouped together by theme along the project timeline. Team members’ perspectives were informed by observations of participants when REVISIT was shared onscreen. We reviewed participant interview transcripts to find relevant excerpts to illustrate our observations.

### Ethical Considerations

The VA Bedford Healthcare System Institutional Review Board determined this work was undertaken to inform VA operations as part of program evaluation and quality improvement activities and was not human subjects research.

## Results

Use of REVISIT was limited to the 16 participants with whom interviews were conducted via VVC. Given the focus on our development of and initial experience with REVISIT, patient perspectives are only included insofar as their observed reactions influenced the team’s experiences and perspectives.

Interviewers used REVISIT to familiarize themselves with relevant details of the index visit prior to conducting interviews. This was particularly helpful when the interviewer did not complete the detailed chart review and was therefore less immersed in the details of each participant’s case or care. REVISIT provided the most salient information at a glance, so interviewers felt it was easier to review than the longer summary extracted from the chart review. With REVISIT, team members also felt better prepared to tailor interview questions to each participant. Additionally, the process of creating or reviewing each participant’s REVISIT visual encouraged the team to consider appropriate language to use during the interviews, such as using “changes in memory” versus “cognitive impairment.”

During the interview, team members used REVISIT as a shared reference point with participants, providing a focus and catalyst for discussion and prompt for further questioning. In one example, REVISIT allowed an interviewer to probe about other aspects of the index visit that were not brought up by the participant organically:

Interviewer: So this [REVISIT] is what we saw as sort of the summary of the visit that you and Mr. XXXX had. We’ve talked about a lot of this. We’ve talked about the changes in diagnoses, the memory changes. It did look like ... they referred you to Audiology to check his hearing. Do you remember that referral at all?

In this way, REVISIT allowed interviewers to bring up contextual details about the visit, which helped to confirm that participants were discussing the index visit. This was particularly important for those who had numerous health care encounters.

Using REVISIT also helped interviewers cross-reference EHR data with participant accounts in real time, confirming congruence or revealing discrepancies between participant recollection and EHR data:

Interviewer: So you mentioned that there were some suggestions for medication changes in the future if anything progresses. We also noticed that there was a recommendation to consider using B12 supplements.

Participant: I don’t recall hearing the recommendation of the B12 supplements.

Interviewer: Okay.

Caregiver: So I didn’t have that in my notes. He has previously been given B12 but it has been quite some time since he’s been given B12 so I don’t remember her mentioning that.

In several cases, interviewers felt that REVISIT seemed to help both veterans and caregivers remember details about the index visit that they otherwise did not bring up:

Caregiver: So, I remember now those conversations and that I had requested – his [the veteran’s] mood was so angry, rage-y – that I had requested Dr. XXXX to increase his quetiapine, get to add one more during the morning and one at noon in addition to the three at night and so your visual helps me remember that.

This contributed to rapport-building by alleviating participants from the onus of remembering every detail of their visit. REVISIT was appreciated by participants, at least one of whom expressed challenges with information recall:

Caregiver: ... it was hard for me to remember what all we talked about that day and that [REVISIT] was very helpful.

However, the use of the tool was not without challenges. Interviewers noted that several participants expressed difficulty seeing REVISIT when shared over the videoconference platform. At least one participant felt that the visual was too light, while several others noted it was too small to read. Most participants who had difficulties with the size of REVISIT viewed it through their cell phone, so the issue of size may partially have to do with the device used:

Participant: I think I can say, for me, it was too small a screen, and you could probably mention, you know, it’s better if you’ve got a tablet or a laptop.

However, one participant on a larger tablet still had issues with font size and readability. It is thus unclear whether these challenges can be attributed to the visual alone or other computer-related factors (eg, whether the VVC window was maximized on the screen and the device’s brightness display). Researchers could not assess or control participants’ computer settings during interviews.

See Table 1 for further organization of interviewer experiences with REVISIT into relevant benefits and challenges.

**Table 1:** Benefits and challenges of using Remembering Healthcare Encounters Visually and Interactively throughout the interview process.

	Interview preparation (development or completion of template)	Interview (data collection)
Benefits	Promoted use of sensitive language (eg, describing symptoms discussed with doctor vs displaying sensitive diagnoses like dementia) Supported organization of participant index visit data from participant’s health records Served as a succinct preinterview refresher for interviewers	Contributed to rapport-building with participants through creation of shared understanding of events Helped catalyze and focus discussion, providing a basis from which the interviewers could probe Appeared to help participants recall and share details about their experiences Allowed interviewers to contrast recall with health record information in real time
Challenges	Required careful thought about displaying sensitive information that may be upsetting to the participants, for example, a new or sensitive diagnosis	Some participants had difficulty seeing visual due to the size or low contrast of the document

## Discussion

### Principal Results

REVISIT is a promising visual tool for enhancing the quality of data collected during interviews with older, rural veterans and caregivers. REVISIT enhanced the interview process by providing a focus and catalyst for discussion and supporting rapport-building with participants. Interviewers felt that the tool supported participants’ recollection of the clinical visit, as many participants noted this while sharing additional details about their experience. Our findings demonstrate that the novel use of visual methodologies during videoconference interviews with older adults is feasible and may be useful in supporting the overall success of qualitative evaluations.

### Comparison With Prior Work

Visual methods, combined with in-depth interviews, have been shown to increase data quality, relevance, and trustworthiness [11,13]. Using REVISIT in our evaluation of GRECC Connect appeared to lead to similar enhancements in data quality by aiding participant recall during interviews, resulting in additional disclosure from participants. This finding is consistent with neuroscience principles that demonstrate how visual stimuli evoke brain regions involved in nonverbal information processing and memory [19].

Our experience is also consistent with other studies that demonstrate visual methods support interviewers in facilitating discussions with participants by prompting further questioning by interviewers, providing direction for discussion, streamlining topic transitions, and promoting increased attention [20-24]. Maintaining participant focus on relevant topics during an interview is essential to generating valid data [25]. Yet, researchers have argued that inhibition, or the ability to direct attention away from irrelevant information, declines with age [5]. Using a simplified visual aid such as REVISIT, which we found to provide a focus for discussion, may be particularly useful when interviewing older adults.

Using REVISIT highlighted, for interviewers, difficulties participants experienced recalling details of their index visit. A real-world implication of this insight is that patients may not remember the health and health care information shared by clinicians to properly care for themselves after the visit. For some evaluation participants, viewing REVISIT was the first time they saw any written information about their visit from a practitioner. There are a number of provider-focused information-giving interventions that have been shown to positively influence patient recall [26,27], including intentional specific structuring of written postdischarge information [28]. At the very least, then, as our experience also suggests, older telemedicine patients may benefit from an after-visit summary outlining pertinent details about their health and health care discussed during the visit.

Consistent with previous studies [23], interviewers felt that using a visual memory aid contributed to rapport-building by providing a shared focus with which to interact and reflect upon. Rapport building is an important dimension of interviewing older adults with communication or cognitive barriers, as these challenges may lead them to view interviewers as threatening and increase feelings of powerlessness or a desire to withdraw from study participation [25,29]. Kirkevold and Bergland [30] suggest allocating more time over the course of a project to establish rapport with older interviewees, which can be challenging for research and evaluation projects with strict time constraints. REVISIT addresses this challenge by providing an accelerated rapport-building option for use directly within participant interviews.

The main challenge of using REVISIT as expressed by participants was the inability of some to see the visual due to its light color and font size. Age-related changes in visual acuity and contrast sensitivity can make it more difficult for older adults to read [5]. Therefore, images should have a high degree of contrast and use a large font. Further, participants in this study noted they or others might benefit from viewing images from a larger screen (eg, a tablet or laptop vs a cell phone). Additionally, although our evaluation of GRECC Connect showed it is possible to use visual tools during video interviews with older adults, it does not address potential barriers to the use of technology among this population; limited knowledge, comfort, or experience with technology, challenges with internet access, and existing cognitive and sensory impairments may hinder participants in studies conducted over videoconferencing platforms [31].

### Additional Considerations

Future users of REVISIT and other similar recall aids should be mindful of how to introduce such tools and integrate them into the interview process. REVISIT may diminish rapport if interviewers share the visual at the wrong time; doing so may inadvertently disrupt the flow of the conversation, distracting participants from the interview as they try to make sense of the visual tool. Using REVISIT also reduces the capacity for nonverbal communication when shared on screen, since this action usually minimizes the window of the participant and researcher across videoconferencing platforms. Additionally, if a participant disagrees with the information presented on the visual, it may create confusion, discomfort, or distrust. Another consideration is the substantial amount of time it takes to prepare the visit summaries and subsequent REVISIT visuals for each interviewee. While the preparation time reduced as the team members gained experience with the methodology, given the time investment needed, this method may not be practical for studies with considerably larger sample sizes.

More research is needed to optimize REVISIT’s usability and understand its acceptability among older adults with cognitive impairments. Future research should also explore the extent to which the visual tool affects recall by systematically comparing appointment recall using REVISIT with interview discussion alone. REVISIT may be useful for understanding the experiences of other patient populations with cognitive impairment (eg, traumatic brain injury and posttraumatic stress disorder) or complex medical care (eg, cancer treatment) and adaptable to in-person use (eg, on an iPad or paper). Further research and evaluation are needed to ensure the efficacy of REVISIT with different populations and settings.

### Limitations

This is a preliminary study. Observations were limited to our sample of 16 veterans and veteran-caregiver dyads, most of whom had some degree of cognitive impairment and were interviewed over VVC, as REVISIT use was only possible through its screen-sharing feature. Further, participants were not systematically asked about their experience of viewing and using REVISIT during the interview. Because of this, we only included patient experiences that directly influenced team members’ own experience with and perceptions of the tool. Additionally, though REVISIT appeared to support recall in this study, it does not guarantee that a participant will truly recall relevant details as opposed to simply agreeing with what they are seeing.

### Conclusions

REVISIT is a novel visual tool that aids the recall of health care encounters by tapping into memories through nonverbal ways of thinking. The use of REVISIT, a carefully curated visual representation of one particular health care encounter, helps to address a number of threats to generating rich, detailed interview data that may be more prevalent when interviewing older adults. As health services research seeks to understand more diverse patient experiences within health care, a tool such as REVISIT may aid recall of health care experiences for those groups for whom it may be more challenging to collect accurate, rich qualitative data. Further research is needed to understand its usefulness with different populations and settings.

## Appendix

Multimedia Appendix 1 Data abstraction template.

## Abbreviations

CVT: Clinical Video Telehealth
EHR: electronic health record
GRECC: Geriatric Research Education and Clinical Center
REVISIT: Remembering Healthcare Encounters Visually and Interactively
RUCA: rural-urban commuting areas
VA: Veterans Health Administration
VVC: VA Video Connect

## References

[1] Kessels RPC (2003). Patients' memory for medical information. J R Soc Med.

[2] McGuire LC (1996). Remembering what the doctor said: organization and adults' memory for medical information. Exp Aging Res.

[3] Anderson JL, Dodman S, Kopelman M, Fleming A (1979). Patient information recall in a rheumatology clinic. Rheumatol Rehabil.

[4] Watson PWB, McKinstry B (2009). A systematic review of interventions to improve recall of medical advice in healthcare consultations. J R Soc Med.

[5] Brown SC, Park DC, Liu LL (2009). How older patients learn medical information. Medical Adherence and Aging: Social and Cognitive Perspectives.

[6] Jones G, Tabassum V, Zarow GJ, Ala TA (2015). The inability of older adults to recall their drugs and medical conditions. Drugs Aging.

[7] Meyer BJF, Russo C, Talbot A (1995). Discourse comprehension and problem solving: decisions about the treatment of breast cancer by women across the life span. Psychol Aging.

[8] Morrow DG, Leirer VO, Carver LM, Tanke ED, McNally AD (1999). Effects of aging, message repetition, and note-taking on memory for health information. J Gerontol B Psychol Sci Soc Sci.

[9] Cohen G, Java R (1995). Memory for medical history: accuracy of recall. Appl Cogn Psychol.

[10] Institute of Medicine, Committee on the Future Health Care Workforce for Older Americans, Board on Health Care Services (2008). Retooling for an Aging America: Building the Health Care Workforce.

[11] Orr ER, Ballantyne M, Gonzalez A, Jack SM (2020). Visual elicitation: methods for enhancing the quality and depth of interview data in applied qualitative health research. ANS Adv Nurs Sci.

[12] Berends L (2011). Embracing the visual: using timelines with in-depth interviews on substance use and treatment. Qual Rep.

[13] Pell B, Williams D, Phillips R, Sanders J, Edwards A, Choy E, Grant A (2020). Using visual timelines in telephone interviews: reflections and lessons learned from the Star Family Study. Int J Qual Methods.

[14] Guerra CE, Schwartz JS, Armstrong K, Brown JS, Halbert CH, Shea JA (2007). Barriers of and facilitators to physician recommendation of colorectal cancer screening. J Gen Intern Med.

[15] Henry SG, Fetters MD (2012). Video elicitation interviews: a qualitative research method for investigating physician-patient interactions. Ann Fam Med.

[16] (2020). VA patient experience journey map. US Department of Veterans Affairs.

[17] Elliot AJ (2015). Color and psychological functioning: a review of theoretical and empirical work. Front Psychol.

[18] Duncan J (2011). The effect of colour and design in labour and delivery: a scientific approach. Opt Laser Technol.

[19] Kandel ER, Schwartz JH, Jessell TM, Siegelbaum SA, Hudspeth AJ, Mack S (2013). Principles of Neural Science, Fifth Edition.

[20] Bagnoli A (2009). Beyond the standard interview: the use of graphic elicitation and arts-based methods. Qual Res J.

[21] Kolar K, Ahmad F, Chan L, Erickson PG (2015). Timeline mapping in qualitative interviews: a study of resilience with marginalized groups. Int J Qual Methods.

[22] Pain H (2012). A literature review to evaluate the choice and use of visual methods. Int J Qual Methods.

[23] Bischof N, Comi A, Eppler MJ (2011). Knowledge visualization in qualitative methods—or how can I see what I say?.

[24] Sheridan J, Chamberlain K, Dupuis A (2011). Timelining: visualizing experience. Qual Res J.

[25] Kvigne K, Gjengedal E, Kirkevold M (2002). Gaining access to the life-world of women suffering from stroke: methodological issues in empirical phenomenological studies. J Adv Nurs.

[26] Lie HC, Juvet LK, Street RL, Gulbrandsen P, Mellblom AV, Brembo EA, Eide H, Heyn L, Saltveit KH, Strømme H, Sundling V, Turk E, Menichetti J (2022). Effects of physicians' information giving on patient outcomes: a systematic review. J Gen Intern Med.

[27] Menichetti J, Lie HC, Mellblom AV, Brembo EA, Eide H, Gulbrandsen P, Heyn L, Saltveit KH, Strømme H, Sundling V, Turk E, Juvet LK (2021). Tested communication strategies for providing information to patients in medical consultations: a scoping review and quality assessment of the literature. Patient Educ Couns.

[28] Ackermann S, Ghanim L, Heierle A, Hertwig R, Langewitz W, Mata R, Bingisser R (2017). Information structuring improves recall of emergency discharge information: a randomized clinical trial. Psychol Health Med.

[29] Domarad BR, Buschmann MT (1995). Interviewing older adults: increasing the credibility of interview data. J Gerontol Nurs.

[30] Kirkevold M, Bergland Å (2009). The quality of qualitative data: issues to consider when interviewing participants who have difficulties providing detailed accounts of their experiences. Int J Qual Stud Health Well-being.

[31] Goldberg EM, Jiménez FN, Chen K, Davoodi NM, Li M, Strauss DH, Zou M, Guthrie K, Merchant RC (2021). Telehealth was beneficial during COVID-19 for older Americans: a qualitative study with physicians. J Am Geriatr Soc.

