# Supplementary material for: The Development and Use of a New Visual Tool (REVISIT) to Support Participant Recall: Web-Based Interview Study Among Older Adults
Source: JMIR Form Res. 2024 Feb 1;8:e52096. doi: 10.2196/52096 (PMC10870211; doi:10.2196/52096)
Supplement: Multimedia Appendix 1 [file formative_v8i1e52096_app1.docx]

**Study ID: _____________**

| **Part 1. Recruitment** | **Answer Options** |
| --- | --- |
| Visit Details - |  |
| Date of Most Recent GRECC Connect visit: |  |
| GRECC Connect provider name/site: |  |
| Primary care provider name/site: |  |
| Chief complaint of current visit: | ☐ Frailty  ☐ Falls  **☐** Cognitive impairment  ☐ Depression  ☐ Palliative Care  ☐ Routine Check In/Follow Up  ☐ Other:__________ |
| Initial reason for referral, if identified: |  |
| Contact Information - |  |
| Primary phone: |  |
| Secondary phone: |  |
| Mailing address: |  |
| Email: |  |
| Rurality Index (From RUCA): |  |
| Caregiver - |  |
| Veteran has an identified caregiver: | ☐ Yes  **☐** No |
| Caregiver details: |  |
| Veteran Attended GRECC Visit with Caregiver or other support: | ☐ Yes  **☐** No |
| Caregiver needs be involved in interview: | ☐ Yes  **☐** No |
| Demographics - |  |
| Age: |  |
| Race: | ☐ White  ☐ Black or African American  ☐ Asian  ☐ Native Hawaiian or Other Pacific Islander  ☐ American Indian or Alaska Native |
| Ethnicity: | ☐ Non-Hispanic/Latinx  ☐ Hispanic/Latinx |
| Sex: | ☐ Male  **☐** Female  ☐ Other |
| Highest level of education: |  |
| Eligibility for interview - |  |
| Eligible? | ☐ Yes  **☐** No  Additional Details: |
| Reasons for exclusion from interview  (check all that apply): | ☐ No GRECC Connect visit found  ☐ Age <65  ☐ Not Rural  ☐ GRECC Connect visit terminated before completion (e.g. unable to participate due to cognitive/hearing impairment and no caregiver to assist)  ☐ Other: ________________ |

| **Part 2. For Interview** | **Answer Options** |
| --- | --- |
| Logistical - |  |
| What is important for the interviewer to know about the patient and/or their abilities?  (not for journey map) |  |
| Initial Referral - |  |
| Is this the Veteran’s first GRECC Connect Visit? | ☐ Yes  **☐** No |
| Reason for initial referral: |  |
| Estimated date of referral: |  |
| Referring provider: |  |
| GRECC Connect Visit - |  |
| Reason for GRECC Connect visit/chief complaint of current visit: | ☐ Frailty  ☐ Falls  **☐** Cognitive impairment  ☐ Depression  ☐ Palliative Care  ☐ Routine Check In/Follow Up  ☐ Other:__________ |
| What was discussed with the PATIENT/CAREGIVER during the visit (for journey map, more lay language): |  |
| Diagnoses (ICD-10 codes) used in GRECC Connect visit: |  |
| Technology challenges noted during visit? | ☐ Yes  **☐** No  Details, if yes: |
| Any comments on access to care in visit note (extract quote(s), no PHI): |  |
| Other Notes from GRECC Connect Visit: |  |
| Context (Services/problems/medications) - |  |
| What is important for the interviewer to know about the patient’s medical history?  (not for journey map) |  |
| Services used ~ 6 months prior to visit:  (check all that apply) | **☐** Visiting nurse  **☐** Day program (may be in SW notes)  **☐** Home-based primary care  **☐** Rehab (PT/OT)  **☐** Social work or case manager  **☐** Respite or caregiver services  **☐** Nutritionist  **☐** Meal support (e.g. Meals on Wheels)  **☐** Home health aide  **☐** Housekeeping support  **☐** Transportation services  **☐** Medical specialty referral (e.g. neurology, cardiology)  **☐** Other: ____________________ |
| New services after visit  (check all that apply) | **☐** Visiting nurse  **☐** Day program (may be in SW notes)  **☐** Home-based primary care  **☐** Rehab (PT/OT)  **☐** Social work or case manager  **☐** Respite or caregiver services  **☐** Nutritionist  **☐** Meal support (e.g. Meals on Wheels)  **☐** Home health aide  **☐** Housekeeping support  **☐** Transportation services  **☐** Medical specialty referral (e.g. neurology, cardiology)  **☐** Other: ____________________ |
| Active problems prior to visit: |  |
| Changes to problem list after visit: |  |
| Active medications prior to visit: |  |
| Changes to medications after visit: (may be in deprescribing note) |  |
